# Short-term improvement of heat tolerance in naturally growing *Acropora* corals in Okinawa

**DOI:** 10.7717/peerj.14629

**Published:** 2023-01-05

**Authors:** Tanya Singh, Kazuhiko Sakai, Jun Ishida-Castañeda, Akira Iguchi

**Affiliations:** 1 Sesoko Station, Tropical Biosphere Research Center, University of the Ryukyus, Motobu, Okinawa, Japan; 2 Graduate School of Engineering and Science, University of the Ryukyus, Nishihara, Okinawa, Japan; 3 Geological Survey of Japan, National Institute of Advanced Industrial Science and Technology, Tsukuba, Ibaraki, Japan; 4 Research Laboratory on Environmentally-Conscious Developments and Technologies [E-code], National Institute of Advanced Industrial Science and Technology, Tsukuba, Ibaraki, Japan

**Keywords:** Acclimatization, *Acropora*, Coral bleaching, Heat stress, Heat tolerance, Naturally growing, Ocean warming

## Abstract

Mass bleaching and subsequent mortality of reef corals by heat stress has increased globally since the late 20th century, due to global warming. Some experimental studies have reported that corals may increase heat tolerance for short periods, but only a few such studies have monitored naturally-growing colonies. Therefore, we monitored the survival, growth, and bleaching status of *Acropora* corals in fixed plots by distinguishing individual colonies on a heat-sensitive reef flat in Okinawa, Japan. The level of heat stress, assessed by the modified version of degree heating week duration in July and August, when the seawater temperature was the highest, was minimally but significantly higher in 2017 than in 2016; however, the same colonies exhibited less bleaching and mortality in 2017 than in 2016. Another study conducted at the same site showed that the dominant unicellular endosymbiotic algal species did not change before and after the 2016 bleaching, indicating that shifting and switching of the Symbiodiniaceae community did not contribute to improved heat tolerance. Colonies that suffered from partial mortality in 2016 were completely bleached at higher rates in 2017 than those without partial mortality in 2016. The present results suggest that either genetic or epigenetic changes in coral hosts and/or algal symbionts, or the shifting or switching of microbes other than endosymbionts, may have improved coral holobiont heat tolerance.

## Introduction

Ocean warming is one of the primary threats to reef-building corals. These corals host endosymbiotic unicellular algae (known as Symbiodiniaceae), which aid host metabolism (LaJeunesse et al., 2018). This symbiotic relationship is extremely susceptible to increases in temperature and irradiance (Fitt et al., 2001). Corals lose their Symbiodiniaceae and undergo bleaching when the sea surface temperature exceeds the local summer maximum even by 1 °C (Muscatine, McCloskey & Marian, 1981; Glynn, 1983; Hoegh-Guldberg, 1999). Further, the frequency and intensity of thermal anomalies have increased over the past few decades, resulting in an increase in regional-scale catastrophic bleaching events (Heron et al., 2016; Hughes et al., 2018; Oliver et al., 2018; Wernberg et al., 2021) that have now started to occur even outside of El Niño conditions (Hughes et al., 2018). Consequently, the likelihood of annual bleaching is expected to increase in the near future.

Certain coral species and genotypes are more resistant to heat stress than others (Loya et al., 2001; Drury, Manzello & Lirman, 2017), but heat-vulnerable coral communities and populations can improve their heat tolerance through adaptation and acclimatization (Buddemeier & Smith, 1999; Coles & Brown, 2003; Putnam, 2021; Brown & Barott, 2022). Coral populations and communities can enhance thermal tolerance through genetic adaptation, which adjusts the frequency of genes coding for fitness-related traits through differential survival and reproduction rates of heat-tolerant genotypes or species (Thompson & van Woesik, 2009; Brown & Cossins, 2011). However, selective adaptation across generations can be constrained by the rapid rate of climate change and the long generation times of corals (Hoegh-Guldberg, 2014). Non-genetic mechanisms allow corals to respond more rapidly to environmental stress and to adjust their tolerance levels (reviewed in Drury, 2020; Putnam, 2021). Such acclimatization processes are rapid, typically lasting for one generation and often reversible (Lämke & Bäurle, 2017). However, as corals are long-lived organisms, acclimatization may still play a critical role in improving their heat resilience. Furthermore, recent studies have suggested that epigenetic changes caused by acclimatization may be inherited by the next generations of corals. Ryu et al. (2018) demonstrated the existence of DNA methylation transmission between adults, sperm, and larvae of the coral *Platygyra daedalea*. Dimond & Roberts (2020) suggested that methylation patterns were partially heritable, with a significant positive correlation between genetic and epigenetic variations in the coral *Porites astreoides*.

Acclimatization enabling corals to survive extreme environmental stress requires prior exposure to sublethal or moderate stress (Brown & Cossins, 2011). This phenomenon, wherein prior stress exposure leads to an enhanced response to subsequent stressors *via* retention of information from the prior exposure, is known as environmental stress memory (Hackerott, Martell & Eirin-Lopez, 2021; Brown & Barott, 2022). Exposure to stress can elicit various responses that are involved in the development of coral resistance to and recovery from heat stress (reviewed in Putnam et al., 2017). In some cases, such responses are maintained even after the temperature returns to normal. Hence, subsequent heat stress exposure of these pre-exposed corals can result in a faster, stronger, and more sensitive response than that of unexposed corals (Brown et al., 2002; Ainsworth et al., 2016; Gintert et al., 2018; Fisch et al., 2019; Hackerott, Martell & Eirin-Lopez, 2021; Brown & Barott, 2022). The capacity for environmental memory depends on various factors, such as the intensity and time between two stress events, and taxon-dependent traits, such as inherent tolerance levels, recovery ability, and capacity for phenotypic plasticity (Hackerott, Martell & Eirin-Lopez, 2021).

Several field and laboratory experiments have provided artificial evidence of coral acclimatization to heat stress, but evidence of natural acclimatization in the field remains scarce. The earliest evidence of acclimatization in nature was observed in *Coelastrea aspera* (previously *Goniastrea aspera*) during the 1995 bleaching event in Phuket, Thailand. The side of the coral colony that was usually exposed to higher solar irradiance was found to be bleached less than the side that was not exposed to light. Both sides of the colonies had identical symbionts, and enhanced thermal tolerance was maintained for 10 years (Brown et al., 2002; Brown et al., 2015). Since then, based on lower bleaching and mortality rates across recurrent bleaching events when thermal anomalies are higher during the second bleaching event, research suggests that coral communities are able to acclimatize to heat stress events (Maynard et al., 2008a; Hughes et al., 2019). However, these studies were not able to account for differential survival by tolerant genotypes or environmental variables such as cloud cover or water movement in reducing bleaching rates in the second bleaching event. Understanding of natural signatures of acclimatization is vital to discern whether the mechanisms observed under experimental conditions are applicable in nature, where corals are simultaneously subjected to multiple stressors. Few studies have provided conclusive evidence for acclimatization to heat stress in nature. One study measured visual bleaching and mortality rates of the same colonies of *Orbicella faveolata* across recurrent bleaching events, one examined stress bands in cores of *Porites* spp., and another artificially induced recurrent annual bleaching in of *P. divaricata* and *O. faveolata* and then transplanted the corals to the field for recovery (Grottoli et al., 2014; Gintert et al., 2018; DeCarlo et al., 2019; Fisch et al., 2019).

We have a limited understanding of how different species acclimatize to recurring annual bleaching events. While there is evidence of acclimatization in nature for some species, prior exposure can also sensitize some species to heat stress, resulting in a more severe response in a recurring event (Brown & Barott, 2022). For example, corals in Hawai’i experienced thermal stress events in 2014 and 2015, and cumulative mortality was highest in 2015 despite some areas showing reduced bleaching (Bahr, Rodgers & Jokiel, 2017). If bleaching reoccurs before the corals can recover from an initial event, they may experience higher mortality. Furthermore, the time required for corals to recover after bleaching varies among species and can also depend on the local environmental conditions (Thomas et al., 2018; Matsuda et al., 2020). Therefore, it is pertinent to identify which and how many species would be able to acclimatize to heat stress when subjected to frequent heat stress events.

Bleaching of heat-sensitive *Acropora* corals, which are key reef-building corals in many Indo-Pacific coral reefs, was observed during the 2016 bleaching event at and around Sesoko Island, Okinawa (Sakai, Singh & Iguchi, 2019; Singh et al., 2019). Individual *Acropora* colonies in fixed plots were used in these studies to visually estimate bleaching as well as post-bleaching mortality and growth rates. Although almost all *Acropora* corals were bleached, approximately 60% of them survived on the southeastern coast of the island (Sakai, Singh & Iguchi, 2019), where severe coral bleaching had occurred in 1998. This subsequent recovery of coral communities has been studied (Loya et al., 2001; van Woesik et al., 2011). Thermal anomalies were also observed at Sesoko Station in 2017. This allowed us to compare the thermal stress response of the same *Acropora* colonies across recurrent bleaching events in 2016 and 2017. We also tested whether moderate heat stress in 2016 enhanced heat tolerance in *Acropora* corals, either through selective adaptation or acclimatization of the coral holobionts.

## Materials and Methods

### Field survey and heat stress assessment

This study was conducted on a well-studied shallow reef flat in front of Sesoko Station, Tropical Biosphere Research Center, University of the Ryukyus, Okinawa, Japan (26°38′N, 127°52′E). We monitored *Acropora* corals in four fixed 2 × 2 m plots in September 2016 and 2017 to compare their bleaching status between these years. The plots were the same as those used in previous studies (Sakai, Singh & Iguchi, 2019; Singh et al., 2019). The plots were located approximately 20 m from the reef edge and were 2 m deep at high tide. We established two subsites approximately 40 m apart and two paired plots approximately 10 m apart within each subsite.

In early September 2016 and 2017, when the bleaching rate was the highest, we captured digital images of the plots from directly above. The plots were divided into four 1 × 1 m subareas by SCUBA diving, and the Canon PowerShot S100 in Canon WP-DC38 underwater housing or Canon S95 in Canon WP-DC43 underwater housing (Canon Inc., Tokyo, Japan) fitted with a wide-angle lens (INON UWL-H100, ×0.60; INON Inc., Kamakura, Japan) were used for imaging. Close-up images of corals were also captured.

Seawater temperature was recorded hourly at the study plots (STSP) by deploying a temperature logger at the study site using the HOBO Onset Water Temperature Pro v2 logger (accuracy: ±0.21 °C from 0 °C to 50 °C) from April 2016 to October 2016 and the HOBO Pendant® Temperature/Light 64 K Data Logger (accuracy: ±0.53 °C from 0 ° C to 50 °C) from October 2016 to October 2017. Heat exposure in 2016 and 2017 were compared by calculating the degree heating week (DHW; °C-week; Liu, Strong & Skirving, 2003) based on the STSP during the hottest months of 2016 and 2017 (July and August) as described by Kumagai, Yamano & Sango-Map-Project C (2018):

$DHW = \displaystyle{1 \over 7}\mathop \sum \limits_{i = 1}^{84} {\rm H}{{\rm S}_{maxi}}{\rm \; if}\text{:}{\rm \; H}{{\rm S}_{\it maxi}}\; \ge {\rm \alpha }^\circ {\rm C}$

where *i*: day, HS_*maxi*_: HotSpots_*maxi*_ = STSP_*i*_ − MMM_*max*_. The overall mean of the monthly means of the STSP in July and August, when the STSP was the highest in 2016 and 2017 (both 29.9 °C), was employed as MMM_*max*_, and α was 1 °C. Traditionally, the DHW is calculated using the long-term summer maxima from the normal years, but we utilized the summer maxima in bleaching years (2016 and 2017) because historical data from the study site was unavailable. Since we used very high values of MMM_*max*_, we expect a low DHW in this study. It is important to note that these DHW values are not representative of traditional DHW values or their corresponding bleaching threshold, *i.e*., bleaching observed at DHW values >4 °C-week and mass bleaching observed at >8 °C-week (Liu, Strong & Skirving, 2003). The purpose of these DHW values is only to compare the heat stress at the same study site between 2016 and 2017. Therefore, we refer to the DHW used in our study as modified degree heating week (mDHW).

Additional thermal indices representing cumulative and acute heat stress, heating rate, and daily temperature range were also estimated for 2016 and 2017 (Table S1). Degree heating days (DHD; °C-days) were calculated by summing the positive deviations of daily mean STSP (STSP_*daily*_) with MMM_*max*_ (Maynard et al., 2008b). The cumulative thermal anomaly (CSA, °C-days) was calculated by performing a trapezoidal integration of daily hotspots (HS_*maxi*_) ≥1 °C during the summer periods each year (Safaie et al., 2018). Degree heating months (DHM; °C-month) were monthly hotspots (MHS) ≥1 °C, summed over the entire year, where MHS is the difference between monthly STSP and MMM_*max*_ (Donner et al., 2005). Acute 1 and acute 2 thermal stressors represented the percentage of days within each year when HS_*maxi*_ ≥1 °C and 2 °C, respectively (Safaie et al., 2018). Max_3_d STSP was the maximum STSP over any three consecutive days during the two warmest months (July and August) (Berkelmans et al., 2004). Heating rate 1 was calculated by dividing DHD by the number of days where STSP exceeded the MMM_*max*_ (Maynard et al., 2008b); heating rate 2 was calculated as the slope of daily STSPs, 3 months before the week with the maximum weekly STSP for both years (Chollett, Enríquez & Mumby, 2014). The difference between the daily maximum and minimum STSP (DTR) was calculated for both the entire year (DTR-total) and for July and August (DTR-heat) (Safaie et al., 2018). The shape of the DTR was estimated by calculating the kurtosis and skew of the daily DTR-total. To compare the light conditions between 2016 and 2017, we used the duration of sunshine (hours per day) in July and August in Nago City, approximately 12 km southwest of the study site (Japan Meteorological Agency, 2022a). Rainfall between 2016 and 2017 was compared using the total precipitation and maximum precipitation (mm) over 1 h and over 10 min in July and August in Motobu town, approximately 5 km northeast of the study site (Japan Meteorological Agency, 2022b).

### Image analysis

All *Acropora* colony species, except very small ones (<3 cm), were identified based on the images in Wallace (1999). Tabular species were clumped as tabular *Acropora* because of the difficulty in identifying Okinawan tabular species based on external morphology (Suzuki et al., 2016). The bleaching status of colonies was visually assessed and classified as unbleached (UB), partially bleached (PB; partially white or the entire colony was pale in color), and completely bleached (CB). We also examined the bleaching trajectories of individual colonies based on their bleaching status in 2016 and 2017 to determine whether the same colony resisted bleaching or showed a reduced degree of bleaching in 2017. The bleaching trajectories are expressed as percentages. The post-bleaching mortality rate of *Acropora* colonies was calculated for April 2016–April 2017 (t1) and April 2017–May 2018 (t2). Whole mortality was scored when the dead skeleton of an entire colony, which was alive at the start of the period, was found at the end of the period. Partial mortality was scored when part of a colony died but a living part remained. The horizontal planar areas (PA) of *Acropora* colonies were measured in April 2016 and 2017, and in May 2018 based on digital images using ImageJ software (Schneider, Rasband & Eliceiri, 2012). The mean diameter (MD) was calculated from the PA, assuming a circular shape for the colony.

The planar colony shape of corals is generally assumed to be elliptical in ecological studies. However, the PA of *Acropora* colonies at our study site was previously found to be similar when the colony shape was assumed to be either elliptical or circular (van Woesik et al., 2011). Growth rates of *Acropora* in time t1 were calculated as the differences in the PA in April 2016 and 2017, divided by the PA in 2016. Growth rates = 0, <1, and >1 indicate that the colony did not grow or shrink, grew negatively due to partial mortality, and grew positively from April 2016 to April 2017, respectively.

### Statistical analyses

Daily differences in STSPs (mean, maximum, and minimum), temperature range (DTR), and precipitation between years were tested using the Friedman test. Differences in the DHW and max_3_d STSP between the years were tested using the Wilcoxon signed-rank test, and the duration of sunshine between the years was tested using the Mann–Whitney U-test. Acute 1 thermal stress was compared between years using McNemar’s test. Acute two thermal stress was not compared statistically because it remained the same between 2016 and 2017. The difference in heating rate (heating rate 2) between years was tested by comparing the slope of daily STSPs 3 months before the week with the maximum weekly STSP (Chollett, Enríquez & Mumby, 2014). These slopes were calculated using a linear regression model. A generalized least square (GLS) linear regression model was constructed with daily STSP as the response variable and “Time (days),” “Year,” and their interaction as explanatory variables. The first-order autoregressive (AR1) correlation structure was incorporated into the model to consider temporal autocorrelation. A significant interaction between “Time” and “Year” would indicate a significant difference in heating rate between the years. Statistical tests could not be performed for thermal indices that had one cumulative value per year. These included DHD, DHM, CSA, heating rate 1, and the shape of the DTR.

For statistical analyses, all *Acropora* present in the study plots were classified into four groups, each of which had a sample size of ≥9 throughout the study period: *A. digitifera*, *A. gemmifera*, tabular *Acropora*, and others (all *Acropora* corals except the dominant groups: *A. digitifera*, *A. gemmifera*, and tabular *Acropora*). Colonies that were alive in both 2016 and 2017 were used to compare the degree of bleaching and partial mortality between the 2 years, while all colonies (dead and alive) were used to compare whole mortality between the 2 years. Therefore, the number of colonies differs depending on the analyses (Table S2). The bleaching degree of a colony was classified either as UB, PB, or CB. Bleaching degree was an ordered nominal variable (UB < PB < CB) and was analyzed using an ordinal regression (McCullagh, 1980). Whole and partial mortality rates were analyzed separately. Whole mortality status of a colony was classified as 1 when it suffered from whole mortality and 0 when it did not suffer from mortality. Similarly, partial mortality status of a colony was classified as 1 when it suffered from partial mortality and 0 when it did not suffer from partial mortality. Partial and whole mortality rates were binary variables and hence were analyzed using a Generalized Linear Model (GLM) with the binomial family of distribution. Initially, a full model was fitted for all tests by including all explanatory variables and their interactions as explanatory variables. The explanatory variables used to compare bleaching degree and mortality rates between 2016 and 2017, were “Colony size” in the preceding April (natural log of MD), “Time”, and “Group”. Interactions between the variables were also added in the full models describing whole and partial mortality rates. We also tested whether any variable could predict bleaching degrees in 2017. For this analysis, “Colony size” in April 2017, growth rates in t1, and “Group” were included as explanatory variables. Additionally, we conducted this analysis for *A. digitifera*, *A. gemmifera*, and tabular *Acropora* separately. Interaction between variables was not added in these models because it resulted in complete separation of response, which in turn resulted in biased statistical inferences. The best-fit model was selected using backward selection. In this approach, we constructed all possible nested models by dropping a single explanatory variable. We then compared nested models with the full model using the log-likelihood ratio test with a significance criterion of *P* < 0.05. A significant difference between a nested model and a full model indicated that the missing variable had a significant effect on the response variable. We repeated this until all explanatory variables had a significant effect on the response variables. Colony ID as a random variable was also included in the best-fit models of the degree of bleaching (both years) and mortality status to account for the repeated measurements of the same colony over time. However, the variance due to colony ID was not significant and did not affect the main results; therefore, it was removed from the final models (Table S3). The assumption of proportional odds for ordinal regression was tested using the Brant test (Brant, 1990) (Table 1).

**Table 1:** Results of the brant test on the proportional odds assumption for the best-fit models describing the degree of beaching in different groups in 2017.

	Groups	Variables	χ	df	Probability
Degree of Bleaching (2016 & 2017)	All *Acropora*	Whole model	2.13	1	0.14
		Time (2017)	2.13	1	0.14
Degree of Bleaching (2017)	All *Acropora*	Whole model	1.3	1	0.26
		Growth	1.3	1	0.26
	Tabular *Acropora*	Whole model	0.0	1	1.00
		Growth	0.0	1	1.00
	*A. digitifera*	Whole model	1.5	1	0.23
		Colony size	1.5	1	0.23

**Note:**

Non-significant *P* values (*P* > 0.05) indicate that the proportional odds assumption holds. *A. gemmifera* was not included because none of the terms were included in the best-fit model. As only one term was included in the best-fit models, the values of the whole model and those of the individual variables (growth or colony size) are the same.

All statistical tests were conducted in R v.3.6.1 (R Core Team, 2019) using the following packages: rcompanion (Mangiafico, 2020), nlme (Pinheiro et al., 2020), lme4 (Bates et al., 2015), MASS (Venables & Ripley, 2002), and brant (Schlegel & Steenbergen, 2020).

## Results

### Degree of heat stress, duration of sunshine and precipitation

**Figure 1:**
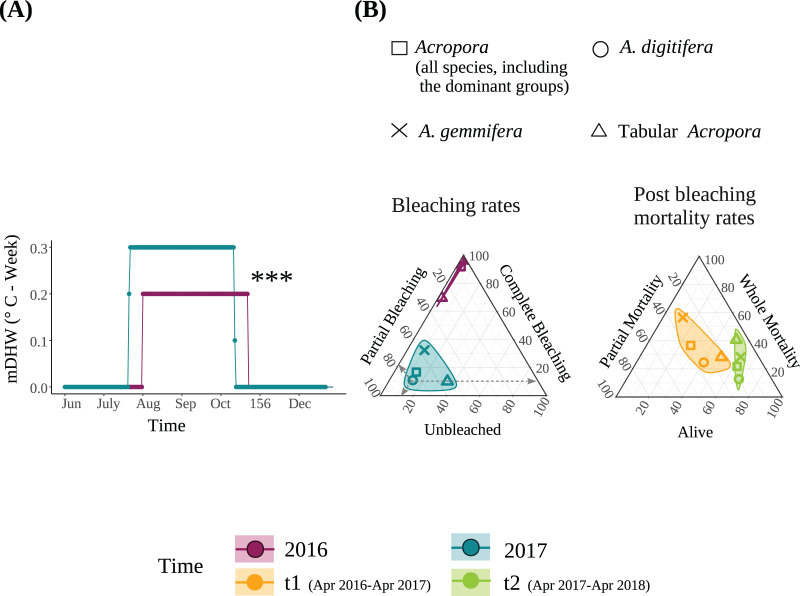
(A) Modified Degree heating week (mDHW) at Sesoko Station reef in 2016 and 2017. Note: The mDHW values in this study are not comparable to the NOAA’s DHW values. (B) Bleaching and post bleaching mortality rates of the dominant *Acropora* groups and all *Acropora* (all *Acropora* colonies, including the dominant groups) present at the study site in 2016 and 2017. Bleaching rates were transformed by adding a value of 1. When using untransformed data, points representing the bleaching rates of 2016 were not visible because <1% were unbleached, and approximately 100% were completely bleached. Asterisks represent significant differences between the years (***P < 0.001).

In our previous study, we showed that corals at the study site were exposed to relatively moderate heat stress in 2016 compared to the extreme heat stress in 1998 (Sakai, Singh & Iguchi, 2019). In this study, we found that the corals at the study site were exposed to similar moderate heat stress (*sensu* Sakai, Singh & Iguchi, 2019) in the summers of 2016 and 2017. Although the mDHW during July and August was significantly higher in 2017 than in 2016 (Fig. 1A), the maximum mDHW in 2016 and 2017 was only 0.2 °C-week and 0.3 °C-week, respectively, and the statistical difference would be unlikely to be biologically relevant. The daily mean SST was 29.88 ± 0.08 °C and 29.98 ± 0.06 °C in 2016 and 2017 (mean ± SE, *N* = 62 per year), respectively, and was significantly different between years (Chi-squared = 5.2, *P* = 0.02). The daily maximum SST was 30.77 ± 0.12 °C and 31.10 ± 0.81 °C in 2016 and 2017 (mean ± SE, *N* = 62 per year), respectively, and was also significantly different between years (Chi-squared = 9.3, *P* = 0.002). Other thermal indices indicated either higher heat exposure in 2017 or similar heat exposure in both years (Figs. S1 and S2, Table S4). These results indicate that the corals were exposed to similar heat stress in 2016 and 2017. The duration of sunshine per day was 7.8 ± 0.4 and 8.7 ± 0.4 h in 2016 and 2017 (mean ± SE, *N* = 62 per year), respectively, and was not significantly different between the years (*Z* = 1.817, *P* = 0.07). Similarly, total precipitation and maximum precipitation over 1 h and over 10 min were not significantly different between years (Table S4).

### Bleaching rate

**Figure 2:**
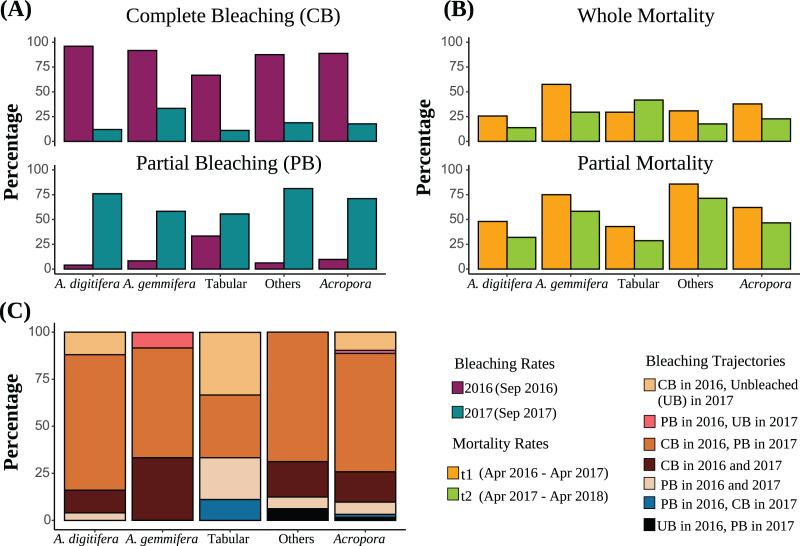
(A) Bleaching rates of the dominant *Acropora* groups (“Others” includes *Acropora* corals other than *A. digitifera*, *A. gemmifera*, and Tabular *Acropora*. “All *Acropora*” includes all *Acropora* corals present in the study, including the dominant groups). Bleaching rates are compared between the same colonies. (B) Post bleaching mortality rates of the dominant *Acropora* groups. Sample numbers are presented above the bars. Partial mortality rates are compared between the same colonies. (C) Bleaching trajectories exhibited by *Acropora* colonies are expressed as percentages.

The detailed bleaching status of *Acropora* corals during the summer of 2016 in the study plots has been reported previously (Sakai, Singh & Iguchi, 2019). Briefly, 6.6% and 92.6% of *Acropora* colonies (*N* = 123) were partially or completely bleached, respectively, on September 3, 2016; only one small colony (1.9 cm in diameter) was not bleached. Tabular *Acropora* (*N* = 18) had a significantly smaller proportion of complete bleaching than *A. digitifera* and *A. gemmifera* (*N* = 39 and 40, respectively; Table 2). In contrast, the bleaching rate of the same *Acropora* colonies decreased significantly in the summer of 2017. The best-fit models describing the degree of bleaching of *Acropora* corals only included the variable “Time”, indicating that the bleaching degree of *Acropora* corals, irrespective of group and colony size, decreased significantly from 2016 to 2017 (*P* < 0.0001, Figs. 1B and 2A, Table 3). The decrease in bleaching degrees from 2016 to 2017 was driven by a significant decrease in the complete bleaching rates and an increase in the partial bleaching rate of *Acropora* colonies in 2017 (Fig. 2A).

**Table 2:** Complete, partial, and total bleaching rates of all *Acropora* colonies present in 2016 and 2017.

Species	Colony size (diameter, cm)	Form	*N*		Complete bleaching		Partial bleaching		Total bleaching	
			2016	2017	2016	2017	2016	2017	2016	2017
*Acropora* (all species)		Mixed	123	62	92.6	17.7	6.6	71.0	99.2	88.7
*A. digitifera*	9.8 ± 0.8	Corymbose	39	25	97.4	12.0	2.6	76.0	100.0	88.0
*A. gemmifera*	16.5 ± 1.4	Digitate	40	12	97.5	33.3	2.5	58.3	100.0	91.6
Tabular	21.2 ± 6.7	Tabular	18	9	72.2	11.1	27.8	55.6	100.0	66.7
*A. intermedia*	9.5 ± 2.4	Arborescent	6	5	83.3	0.0	16.7	100.0	100.0	100.0
*A. robusta*	26.3	Arborescent	1	1	100.0	0.0	0.0	100.0	100.0	100.0
*A. monticulosa*	25.2 ± 4.9	Digitate	7	3	100.0	33.3	0.0	66.7	100.0	100.0
*A. valida*	8.8 ± 1.7	Corymbose	3	3	100.0	0.0	0.0	100.0	100.0	100.0
*A. aspera*	18	Arborescent	1	1	100.0	100.0	0.0	0.0	100.0	100.0
*A. humilis*	11.7 ± 3.7	Digitate	2	1	100.0	100.0	0.0	0.0	100.0	100.0
*A. latistella*	15.3	Corymbose	1	1	100.0	0.0	0.0	100.0	100.0	100.0
Unknown	1.9	Unknown	1	1	100.0	0.0	0.0	100.0	100.0	100.0

**Note:**

The mean colony size shown is that of April 2016 (mean ± SE).

**Table 3:** Results of the backward selection for the models describing the bleaching degree and mortality rates.

Response	Groups	Variables	*N*	df	LRT	Pr (>Chi)
Bleaching degree (2016 & 2017)*	All *Acropora*	“Colony Size”	121	1	0.8	0.38
		“Group”		3	6.8	0.08
		“Time”		1	68.2	***<0.00001***
Bleaching degree (2017)*		“Group”	59	3	3.0	0.38
	All *Acropora*	“Growth”		1	7.0	***0.01***
		“Colony Size”		1	1.0	0.32
	*A. digitifera*	“Growth”	25	1	0.4	0.53
		“Colony Size”		1	9.3	***0.002***
	*A. gemmifera*	“Growth”	23	1	0.5	0.49
		“Colony Size”		1	0.1	0.75
	Tabular *Acropora*	“Growth”	9	1	16.9	***0.00004***
		“Colony Size”		1	0.00	1
Partial mortality	All *Acropora*	“Colony Size” × “Time” × “Group”	113	3	5.2	0.16
		“Group” × “Time”		3	2.3	0.51
		“Colony Size” × “Group”		3	5.7	0.13
		“Colony Size” × “Time”		1	18.2	***0.0004***
		“Group”		3	5.6	***0.02***
Whole mortality	All *Acropora*	“Colony Size” × “Time” × “Group”	193	3	0.7	0.87
		“Colony Size” × “Group”		3	2.3	0.51
		“Group” × “Time”		3	3.1	0.38
		“Colony Size” × “Time”		1	1.3	0.26
		“Colony Size”		1	0.03	0.87
		“Time”	196	1	3.9	0.05
		“Group”		3	12.3	***0.01***

**Note:**

* Interactions between the explanatory variables were not included in the bleaching degree analyses because they caused technical issues (complete separation) in the model.

The variables selected in the final models have significant *P* values and are indicated in bold. Two models, with and without the terms of interest, were compared using a log-likelihood ratio test. If the *P* values were >0.05, the terms were eliminated from the model. *N* is the number of observations for each model. df is the degree of freedom, which equals to the difference in the number of parameters between the models being compared. LRT is the log-likelihood ratio based on the difference in the residual deviance of the two models. Significant *P* values (<0.05) are indicated in italics and bold.

Of *Acropora* (all *Acropora* corals present in the study plots), *A. digitifera*, *A. gemmifera*, tabular *Acropora*, and other colonies, 62.9%, 72.0%, 58.3%, 33.3%, and 68.8% were bleached completely in 2016 and partially in 2017, respectively (Fig. 2C). Further, 9.7%, 12.0%, and 33.3% of *Acropora*, *A. digitifera*, and tabular *Acropora* colonies were bleached completely in 2016 but remained unbleached in 2017, respectively (Fig. 2C).

**Figure 3:**
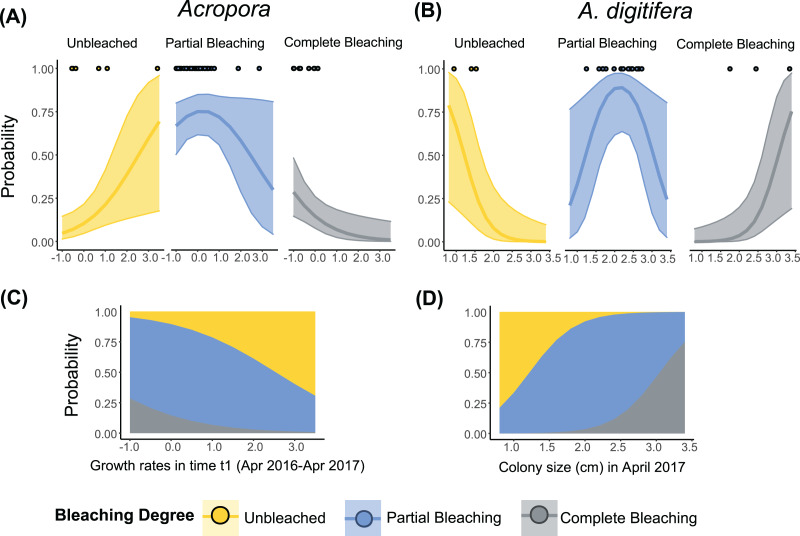
Relationship between the degree of bleaching in 2017 and the growth rates or colony size of *Acropora* (all *Acropora* present in the study, including the dominant groups) and *A. digitifera*. Tabular *Acropora* and *A. gemmifera* are not shown because neither growth nor colony size had a significant effect on bleaching degree. Lines in (A) and (B) represent ordinal regression fit with 95% confidence intervals. Dots in (A) and (B) represent the observed data. Stacked area plots showing the probability of different levels of bleaching are presented in (C) and (D).

The best-fit models, describing the degree of bleaching in 2017 for *Acropora* corals included the term “Growth rates” (Table 3). The degree of bleaching in 2017 for *Acropora* corals had a negative relationship with the “Growth rates” in time t1 (Table S5, Fig. 3B). Colonies that had zero or negative growth rates in t1 bleached more severely in 2017 compared to colonies with positive growth rates in t1. For example, the probability of being completely bleached was highest for *Acropora* colonies that suffered from partial mortality in t1 (Figs. 3A and 3C). In contrast, the probability of being unbleached was the highest for *Acropora* colonies with positive growth rates in t1 (Fig. 3A).

The best-fit models describing the degree of bleaching in 2017 for *A. digitifera* only included the term “Colony size” (Table 3). The degree of bleaching in 2017 for *A. digitifera* had a negative relationship with “Colony size” in April 2017 (Table S5, Fig. 3B). Larger colonies of *A. digitifera* bleached more severely in 2017 compared to the smaller colonies. Therefore, as the colony size of *A. digitifera* increased, the probability of being unbleached decreased, while that the probability of being completely bleached increased (Figs. 3B and 3D). The probability of partial bleaching was higher for intermediate-sized *A. digitifera* colonies (Figs. 3B and 3D).

### Mortality rate

The best-fit model describing whole mortality only included the variable “Group” (*P* = 0.01, Table 3). The variable “Time” was removed from the model because it only had a marginal effect on whole mortality rates (*P* = 0.05, Table 3). Although whole mortality rates of *Acropora*, *A. digitifera*, *A. gemmifera*, and others decreased from t1 (April 2016–April 2017) to t2 (April 2017–May 2018), the differences were only marginal (Fig. 2B, Table 3). Mortality rates of *A. gemmifera* were significantly higher than those of *A. digitifera* (adjusted *P* = 0.006). Pairwise differences among other groups were not significant (adjusted *P* range 0.1–0.9).

The best-fit model describing the partial mortality included the terms “Group” and the interaction between “Colony size” and “Time” (*P* = 0.02 and 0.0004, Table 3). The effect of “Colony size” on partial mortality rates for *Acropora* was significant only in t1, where the probability of partial mortality increased with “Colony size” (adjusted *P* = 0.004; Fig. S3). The partial mortality rates of small and average-sized colonies of *Acropora* were similar in t1 and t2 (adjusted *P* = 0.06 and 0.8), whereas partial mortality of larger-sized colonies of *Acropora* was significantly higher in t1 than in t2 (adjusted *P* = 0.03). Partial mortality of other *Acropora* was significantly higher than that of *A. digitifera* and tabular *Acropora* (adjusted *P* = 0.004 and 0.04). Partial mortality rates of *A. gemmifera* were marginally higher than those of *A. digitifera* (adjusted *P* = 0.05). Pairwise differences among other *Acropora* groups were not significant (adjusted *P* range 0.2 to 0.8).

## Discussion

Our study highlights the importance of field studies that differentiate between individual coral colonies and show that reef corals can improve their heat-stress tolerance in the face of climate change through acclimatization. Some field studies had reported improved heat-stress tolerance of reef corals in repeatedly surveyed coral assemblages when thermal stress was equivalent or higher during the second bleaching event, but without distinguishing individual colonies (Pratchett et al., 2013; McClanahan & Muthiga, 2014; McClanahan, 2017; Hughes et al., 2019). In these studies, adaptation, *i.e*., the selection of heat-stress tolerant genotypes, and acclimatization through phenotypic plasticity are the possible mechanisms for increased heat-stress tolerance in corals (Pandolfi et al., 2011). By repeatedly monitoring individual coral colonies in the field using permanent plots (Gintert et al., 2018) or by tagging colonies (Grottoli et al., 2014; Dilworth et al., 2021; although colonies were transplanted), acclimatization can be distinguished from adaptation by natural selection in a natural environment.

In our study, *Acropora* colonies that were bleached but survived moderate heat stress in 2016 (Sakai, Singh & Iguchi, 2019) did not show the same effects under similar heat stress in 2017. Besides temperature, several environmental factors such as lower light intensity, higher wind speed, higher water flow, and typhoons can also mitigate coral bleaching severity (Fitt & Warner, 1995; Nakamura & van Woesik, 2001; Smith, 2001; West & Salm, 2003). However, in our study, the light and precipitation regime, wind speed, wave height, number of cloudy days, and the timing and frequency of typhoons around the study site were found to be similar in the summer of 2016 and 2017 (Table S4; Supplemental Data in Singh et al., 2022). This indicates that the improved heat-stress tolerance was not caused by adaptation or better environmental conditions in 2017, but by the acclimatization of the coral holobionts. The results obtained in our study are similar to those reported by Gintert et al. (2018), who demonstrated that the same colonies of *O. faveolata* bleached less severely during the second bleaching event, even when the temperatures were higher. However, our study revealed the acclimatization of naturally growing *Acropora*, an important coral genus in Indo-Pacific reefs, including Okinawa.

A moderate thermal stress event in 2016, in combination with higher daily temperature variations in 2017, may have enabled *Acropora* corals in our study to acclimatize to heat stress. Widespread and recurrent annual bleaching was observed globally from 2014 to 2017 (Eakin, Sweatman & Brainard, 2019). Acclimatization to heat stress was observed in some cases, while in others recurrent annual bleaching resulted in severe cumulative mortality without any evidence of acclimatization (Bahr, Rodgers & Jokiel, 2017; Raymundo et al., 2019; Ritson-Williams & Gates, 2020). Such contrasting trajectories following the initial stress exposure could be due to the differences in coral community composition, intensity, duration, and return time of thermal stress events, and local environmental conditions (Brown & Barott, 2022). For example, consecutive bleaching events in Hawai’i in 2014 and 2015 led to significant cumulative mortality of corals (Bahr, Rodgers & Jokiel, 2017; Ritson-Williams & Gates, 2020). These heat stress events were of extreme intensity (DHW > 6 °C-week) and were only 10 months apart, which was probably not enough time for corals to recover and acclimatize (Matsuda et al., 2020). In contrast, the 2016 heat stress event at our study site was moderate in intensity, which might have allowed *Acropora* corals to recover and acclimatize to heat stress within a year. We assume that, had the *Acropora* corals at our study site been exposed to more extreme temperatures, they might have also died or failed to recover within a year. The extreme thermal stress event in 1998 (DHW = 10.6 °C-week) at the same study site caused nearly 100% mortality of *Acropora* corals (Loya et al., 2001; Sakai, Singh & Iguchi, 2019). Apart from the moderate heat stress event in 2016, the daily temperature ranges in 2017 were significantly higher than those in 2016 (Table S4, Fig. S3). Daily extreme temperature fluctuations often expose corals to temperatures above their local bleaching thresholds for a short enough time to induce acclimatization and avoid mortality (Oliver & Palumbi, 2011; Barshis et al., 2013; Kenkel & Matz, 2017; Safaie et al., 2018). The daily temperature range in our study (~1.5 °C) is much lower than in the studies where acclimatization was induced by fluctuating temperatures (5–10 °C) (Palumbi et al., 2014; Camp et al., 2018). However, a combination of sublethal stress in the summer of 2016 and a higher daily temperature range in 2017 may have enabled the *Acropora* holobionts in our study to acclimatize to heat stress.

Prior exposure to sublethal heat stress can enhance the heat tolerance of corals through several mechanisms. Association between the microbiome (unicellular dinoflagellates of the family Symbiodinaceae, bacteria, archaea, fungi, and viruses) and the host coral colony may not always be permanent. Therefore, corals can enhance their heat tolerance by expelling the already present heat-vulnerable symbionts and acquiring novel heat-tolerant types (“switching”), or by repopulating the remaining heat-tolerant types (“shuffling”) (Buddemeier & Fautin, 1993; Baker, 2003). Similarly, shifts in other microbial assemblages have been reported to enhance the thermal tolerance of corals (Reshef et al., 2006; Bourne et al., 2008; Bourne, Morrow & Webster, 2016; Ziegler et al., 2017; Rosado et al., 2019). Moderate heat stress can also cause a constitutive upregulated expression of genes involved in defense against heat stress, like the production of heat shock proteins and antioxidant enzymes, and the regulation of programmed cell death, even in the absence of heat stress (Barshis et al., 2013; Palumbi et al., 2014; Bay & Palumbi, 2015). Furthermore, corals can also respond to heat stress by modifying the nature of DNA molecules (*e.g*., through DNA methylation) and chromatin-associated proteins (histone methylation and acetylation) without changing the genetic code. Such epigenetic mechanisms can increase thermal tolerance by enhancing the activation of genetic stress responses (Eirin-Lopez & Putnam, 2019). An increase in heat tolerance through epigenetic changes can occur in both host corals and/or endosymbionts (Putnam, Davidson & Gates, 2016; Liew et al., 2018; Yang, Li & Lin, 2020). These possible mechanisms for improving heat-stress tolerance in *Acropora* corals may not be mutually exclusive; future studies are needed to examine these mechanisms in a multidirectional manner.

The readjustment of Symbiodiniaceae composition is one possible mechanism of acclimatization, but this was not the case for the increased heat-stress tolerance of *A. digitifera* at our study site. The Symbiodiniaceae composition of *A. digitifera* located next to our study plots was compared before and 2 months after the 2016 bleaching event at the same site (J. Ishida-Castañeda, 2016, unpublished data). These results showed that the types of Symbiodiniaceae present in the corals changed significantly before and after the 2016 moderate heat stress, but the dominant type remained the same. Both before and after the moderate heat stress in 2016, the dominant Symbiodiniaceae type in *A. digitifera* (ITS2 type) was the same as that of C50a/C50c-C3-C3b, except for one colony (C50a, 160908_06). Although other endosymbionts had changed, their relative abundance was <0.1% and they all belonged to the genus *Cladocopium*. This indicates that significant “shuffling” and “switching” of Symbiodiniaceae did not occur because of moderate heat stress. Although endosymbiont types were not examined in other species, it is likely that shuffling or switching of Symbiodiniaceae did not occur in other species, as indicated by previous research (Goulet & Coffroth, 2003; Stat et al., 2009; Coffroth et al., 2010; Smith et al., 2017; Dilworth et al., 2021). However, Jung et al. (2021) showed that the changes in the background Symbiodiniaceae community composition could sometimes influence heat tolerance in *Acropora*.

Basal energy reserve levels of corals may play an essential role in facilitating rapid acclimatization to repeated heat-stress events (Grottoli et al., 2014). Bleaching significantly lowers the energy reserves of corals (Anthony, Connolly & Hoegh-Guldberg, 2007; Rodrigues & Grottoli, 2007; Leinbach et al., 2021). The extent of the loss in energy reserves and the ability to recover the energy after the heat exposure is determined by the intensity of and the time between the two consecutive heat stress events, heterotrophic plasticity, and local environment (Grottoli, Rodrigues & Palardy, 2006; Rodrigues & Grottoli, 2007; Anthony et al., 2009; Raymundo et al., 2019; Sale et al., 2019). Furthermore, if any other processes that utilize energy coincide with the bleaching event, corals can become more vulnerable and potentially less able to acclimatize to heat stress. For example, failure to acclimatize to a simulated consecutive bleaching event was linked to low basal energy reserves, which in turn was attributed to the peak in temperatures coinciding with end of the reproductive season (Grottoli et al., 2014; Schoepf et al., 2015). *Acropora* corals at our study site generally spawn synchronously in June, a month before the temperatures begin to rise (Baird et al., 2022; Singh et al., 2022). Although we do not know if this is enough time for *Acropora* corals to recover their basal energy reserves after reproduction, the reproductive period does not overlap with increased heat stress at our study site. Furthermore, the bleaching event in the summer of 2016 may have also reduced the energy levels of the *Acropora* corals. Colonies acclimatized to heat stress may have been the ones that could recover their energy levels by the time temperatures peaked in 2017. Future studies should examine the time required for various *Acropora* species to restore their energy reserves after spawning at our site to test if reproductive cycles can influence bleaching resilience and acclimatization potential.

Growth rates after the 2016 bleaching event could explain the variations in the degree of bleaching among *Acropora* corals in 2017. In our study, the colonies that had negative growth rates from April 2016 to April 2017 bleached more severely than the colonies that had positive growth rates (Fig. 3). The depressed growth rates in some of these colonies during this period were most likely due to the heat stress event that occurred in the summer of 2016 (July–September). During the moderate stress event in 2016, almost all *Acropora* colonies had bleached completely. Out of these, the colonies suffering from partial mortality bleached more severely in 2017 than those which did not (Sakai, Singh & Iguchi, 2019). Both bleaching and partial mortality can significantly lower coral energy reserves (Piñón-González & Banaszak, 2018; Leinbach et al., 2021). Therefore, *Acropora* colonies that suffered from partial mortality following the 2016 bleaching event likely had low basal energy levels, which would have made them more vulnerable to bleaching during the 2017 heat stress event (Anthony, Connolly & Hoegh-Guldberg, 2007; Rodrigues & Grottoli, 2007; Grottoli et al., 2014). However, future studies should test the link between energy reserves, growth rates, and bleaching susceptibility in a controlled experiments.

## Conclusions

This study highlighted that periodic field surveys of the same individual coral colonies can be a powerful tool for identifying the signatures associated with heat-resilient corals in their natural environment. Our results indicate that naturally growing *Acropora* corals improved their heat tolerance over the duration of one year after experiencing moderate heat stress one summer. This may be due to a variety of factors, *i.e*., genetic or epigenetic changes in the coral hosts and/or the algal symbionts, or shifting or switching of the microbial composition.

## Supplemental Information

10.7717/peerj.14629/supp-1 Supplemental Information 1 Supplemental Figures and Tables.

10.7717/peerj.14629/supp-2 Supplemental Information 2 Raw data.

## Additional Information and Declarations

### Data Availability

The following information was supplied regarding data availability: The raw data of corals and seawater temperatures and the summarized data for corals are available in the Supplemental File.
