# Supplementary material for: Short-term improvement of heat tolerance in naturally growing Acropora corals in Okinawa
Source: PeerJ. 2023 Jan 5;11:e14629. doi: 10.7717/peerj.14629 (PMC9826613; doi:10.7717/peerj.14629)
Supplement: Supplemental Information 1 [file peerj-11-14629-s001.docx]

Supplementary Material

**Supplementary Tables**

**Supplementary Table S1.** Thermal indices used in this study. Abbreviations are mentioned in parenthesis, and units are noted in square brackets.

| **Indices** | **Description** | **Reference** |
| --- | --- | --- |
| **Cumulative stress** | | |
| Degree heating days (DHD)  [°C -days] | The value is the summed positive deviations of daily mean sea surface temperatures (STSP*_daily_*) from the mean of the monthly means of STSP in July and August (MMM*_max_*)  .  $DHD= \sum({STSP}_{daily}-MMMmax), \mathrm{if}: \left( {STSP}_{daily}-MMMmax \right)>0$  Single cumulative value per year were calculated. | Maynard et al. (2008) |
| Cumulative thermal anomaly  (CSA )  [°C -  days] | Trapezoidal integration of daily hotspot (HS*_maxi_*) ≥ 1°C, during summer periods for each year. Where daily HS*_maxi_* is the difference between the STSP*_daily_* and MMM*_max_*.  Single cumulative value per year were calculated. | Safaie et al. (2018) |
| Degree heating week (DHW)  [°C -week] | Cumulative thermal stress over a 12-week window.  $DHW= \frac{1}{7}\sum_{i=1}^{84} ({HSmax}_{i}, if: {HSmax}_{i}\geq\alpha^{\circ}C)$  $Where: \alpha=1 ^{\circ}C$  Daily DHW values were calculated. | Liu, Strong & Skirving (2003) |
| Degree heating month (DHM)  [°C - month] | Monthly hotspots (MHS) ≥ 1°C, summated over the entire year. Where MHS is the difference between monthly STSP and MMM*_max_*.  $DHM=\sum_{i=1}^{i=12} ({MHS}_{i}, {if: MSH}_{i} \geq1 ^{\circ}C)$  Single cumulative value per year were calculated. | Donner et al. (2005) |
| **Acute stress** | | |
| Acute 1 | Presence or absence of days when HS*_maxi_*≥ 1°C within each year.  Single cumulative value expressed as percentage of days when HS*_maxi_* ≥ 1°C within each year were calculated. | Safaie et al. (2018) |
| Acute 2 | Presence or absence of days when HS*_maxi_* ≥ 2°C within each year.  Single cumulative value expressed as percentage of days when HS*_maxi_* ≥ 2°C within each year were calculated. |  |
| Max_3_d STSP [°C] | Maximum STSP over any consecutive three days during the warmest two months (July-August).  Daily Max_3_d STSPs from July-August were estimated. | Berkelmans et al. (2004) |
| **Rate in change of STSP** | | |
| Heating rate 1  [°C/day] | DHD divided by the number of days were STSP exceeded long- term summer mean. | Maynard et al. (2008b) |
| Heating rate 2  [°C/day] | Slope of daily STSPs three months prior to the week with maximum weekly STSP for both years | Chollett, Enríquez & Mumby (2014) |
| **High frequency STSP variability** | | |
| Daily temperature variation  [°C] | Difference between daily maximum and daily minimum STSP  DTR total: Mean DTR over entire year.  DTR heat: Mean DTR over warmest two months in the year (July - August) | Safaie et al. (2018) |
| DTR shape | Kurtosis of daily DTR values of each year.  Skewness of daily DTR values of each year | Safaie et al. (2018) |

**Supplementary Table S2.** The sample number used for bleaching and mortality analyses for each group in each year. The group “Others” includes all *Acropora* except *A. digitifera*, *A. gemmifera*, and Tabular *Acropora.* The group “Acropora” includes all *Acropora* present in the study plots.

| **Response** | **Groups** | **2016** | **2017** |
| --- | --- | --- | --- |
| Bleaching (Complete and Partial) | *A. digitifera* | 25 | 25 |
|  | *A. gemmifera* | 12 | 12 |
|  | Tabular *Acropora* | 9 | 9 |
|  | Others | 16 | 16 |
|  | *Acropora* | 62 | 62 |
| Whole Mortality | *A. digitifera* | 39 | 29 |
|  | *A. gemmifera* | 40 | 17 |
|  | Tabular *Acropora* | 17 | 12 |
|  | Others | 26 | 17 |
|  | *Acropora* | 122 | 75 |
| Partial Mortality | *A. digitifera* | 25 | 25 |
|  | *A. gemmifera* | 12 | 12 |
|  | Tabular *Acropora* | 7 | 7 |
|  | Others | 14 | 14 |
|  | *Acropora* | 58 | 58 |

**Supplementary Table S3.** Effect of random variable, Colony ID on the best-fit models describing the variations in bleaching degree and mortality rates of all *Acropora* corals in 2016 and 2017. Models with and without the random effect were compared using a log-likelihood ratio test. If the p values were > 0.05 the random effect was eliminated from the model. df is the degree of freedom, which equals the difference in the number of parameters between the models being compared. LRT is the log likelihood ratio based on the difference in the residual deviance of the two models.

| **Response** | **df** | **LRT** | **Pr(>Chisq)** |
| --- | --- | --- | --- |
| Bleaching Degree (2016 & 2017) | 1.0 | 0.0 | 1.0 |
| Partial Mortality | 1.0 | 1.2 | 2.7 |
| Whole Mortality | 1.0 | 1.4 | 0.2 |

**Supplementary Table S4.** Results of statistical tests that compared various variables or thermal indices and the duration of sunshine between 2016 and 2017. Significant P values (< 0.05) are indicated in italic and bold font. Thermal indices are shown in Table S1.

| **Test** | **Variables** | **Chi-squared** | **p value** |
| --- | --- | --- | --- |
| **Friedman test** | Daily Minimum STSP | 0.1 | 0.79 |
|  | Daily Mean STSP | 5.2 | ***0.02*** |
|  | Daily Maximum STSP | 9.3 | ***0.002*** |
|  | DTR_total_ | 5.5 | ***0.02*** |
|  | DTR_h_ | 10.9 | ***0.001*** |
|  | Total precipitation (mm) | 2.9 | 0.09 |
|  | Maximum precipitation over 1 hour | 3.5 | 0.06 |
|  | Maximum precipitation over 10 minutes | 2.5 | 0.18 |
| **Wilcoxson signed**  **rank test** |  | **V statistics** |  |
|  | Max3_d_STSP | 787.0 | 0.19 |
|  | DHW | 874.0 | ***<0.00001*** |
| **McNemar's test** |  | **Z statistics** |  |
|  | Acute1 | -1.0 | 0.32 |
| **GLS regression** |  | **Value** |  |
|  | Heating rate (GLS Slope) | -0.93 | 0.40 |
| **Mann-Whitney U-test** |  | **Z- statistics** |  |
|  | Duration of sunshine | 1.8, | 0.07 |

**Supplementary Table S5.** Summary of best fit models describing bleaching degree of *Acropora* corals. There are two intercepts per model because it is an ordinal regression. UB|PB means log odds of being unbleached versus being partially or completely bleached. PB|CB means log odds of being unbleached or partially bleached compared to completely bleached. Significant P values (< 0.05) are indicated in italic and bold font. UB, unbleached; PB, partially bleached; CB, completely bleached.

| **Response** | **Group** | **Model Summary** | **Terms** | **Value** | **Std. Error** | **t value** | **p value** |
| --- | --- | --- | --- | --- | --- | --- | --- |
| **Bleaching Degree (2016 & 2017)** | All *Acropora* | Coefficients | Time - 2017 | -3.6 | 0.5 | -6.8 | ***<0.00001*** |
|  |  | Intercepts | UB\|PB | -5.5 | 0.6 | -9.1 | ***<0.00001*** |
|  |  |  | PB\|CB | -2.0 | 0.4 | -5.1 | ***<0.00001*** |
| **Bleaching Degree (2017)** | All *Acropora* | Coefficients | Growth | -0.8 | 0.4 | -2.4 | ***0.02*** |
|  | *A. digitifera* | Coefficients | Colony Size | 3.2 | 1.3 | 2.5 | ***0.01*** |
|  | Tabular *Acropora* | Coefficients | Growth | -75.5 | 226650 | 0 | 1.0 |
|  | All *Acropora* | Intercepts | UB\|PB | -2.2 | 0.4 | -4.9 | ***<0.00001*** |
|  |  | Intercepts | PB\|CB | 1.8 | 0.4 | 4.8 | ***<0.00001*** |
|  | *A. digitifera* | Intercepts | UB\|PB | 3.8 | 2.2 | 1.7 | 0.08 |
|  |  | Intercepts | PB\|CB | 9.6 | 3.3 | 2.9 | ***0.004*** |
|  | Tabular *Acropora** | Intercepts | UB\|PB | -28.9 | 100381.1 | 0 | 1.0 |
|  |  | Intercepts | PB\|CB | 51.9 | 247807.4 | 0 | 1.0 |

**Supplementary Table S6.** Model summaries of best fit models describing whole or partial mortality of all *Acropora*. Significant P values (< 0.05) are indicated in italic and bold font.

| **Response** | **Model variables** | | | | |
| --- | --- | --- | --- | --- | --- |
|  | **Terms** | **Estimate** | **Std. Error** | **z value** | **Pr(>\|z\|)** |
| **Whole Mortality** | Intercept | -1.0 | 0.4 | -3.0 | 0.003 |
|  | Group *A. digitifera* | -0.3 | 0.5 | -0.7 | 0.5 |
|  | Group *A. gemmifera* | 1.0 | 0.4 | 2.3 | 0.02 |
|  | Group Tabular | 0.4 | 0.5 | 0.8 | 0.5 |
| **Partial Mortality** | Intercept | -3.0 | 1.6 | -1.9 | 0.06 |
|  | Colony Size | 2.3 | 0.7 | 3.1 | 0.002 |
|  | Time t2 | 3.4 | 1.8 | 1.9 | 0.06 |
|  | Group *A. digitifera* | -2.3 | 0.7 | -3.4 | 0.001 |
|  | Group *A. gemmifera* | -0.7 | 0.8 | -1.0 | 0.3 |
|  | Group Tabular | -2.5 | 0.9 | -2.7 | 0.008 |
|  | Colony Size - Time t2 | -1.7 | 0.8 | -2.1 | 0.03 |
|  | **Summary of deviance residuals** | | | | |
|  | **Min** | **1Q** | **Median** | **3Q** | **Max** |
| **Whole Mortality** | -1.2 | -0.8 | -0.7 | 1.2 | 1.8 |
| **Partial Mortality** | -2.2 | -0.9 | 0.2 | 0.9 | 1.9 |
|  | **Deviance** | | | | |
|  | **Null** | |  | **Residual** | |
| **Whole Mortality** | 246.15 on 195 Df | |  | 233.82 on 192 df | |
| **Partial Mortality** | 155.15 on 112 Df | |  | 116.68 on 106 Df | |

**Supplementary Figures**


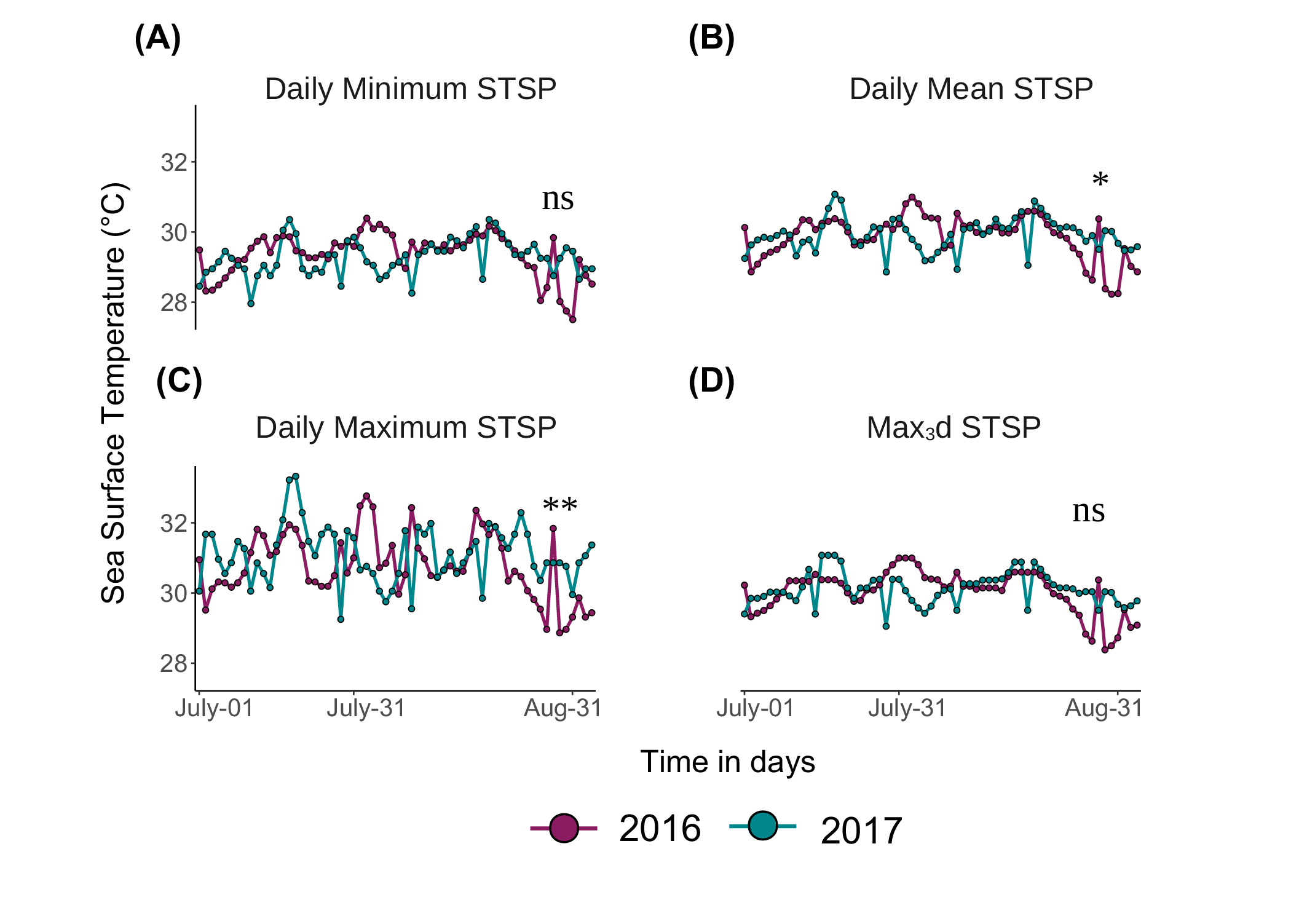
 **Supplementary Figure S1.** (A) Daily minimum, (B) mean, (C) maximum, and (D) Max_3_d (maximum over any consecutive three days) STSPs (seawater temperature recorded hourly at the study plots) during the warmest two months. Asterisks represent significant differences between years or time periods (‘***’ P < 0.001, ‘**’ P < 0.01 ‘*’ P < 0.05 ‘·’ ‘ns’ P > 0.05).

**
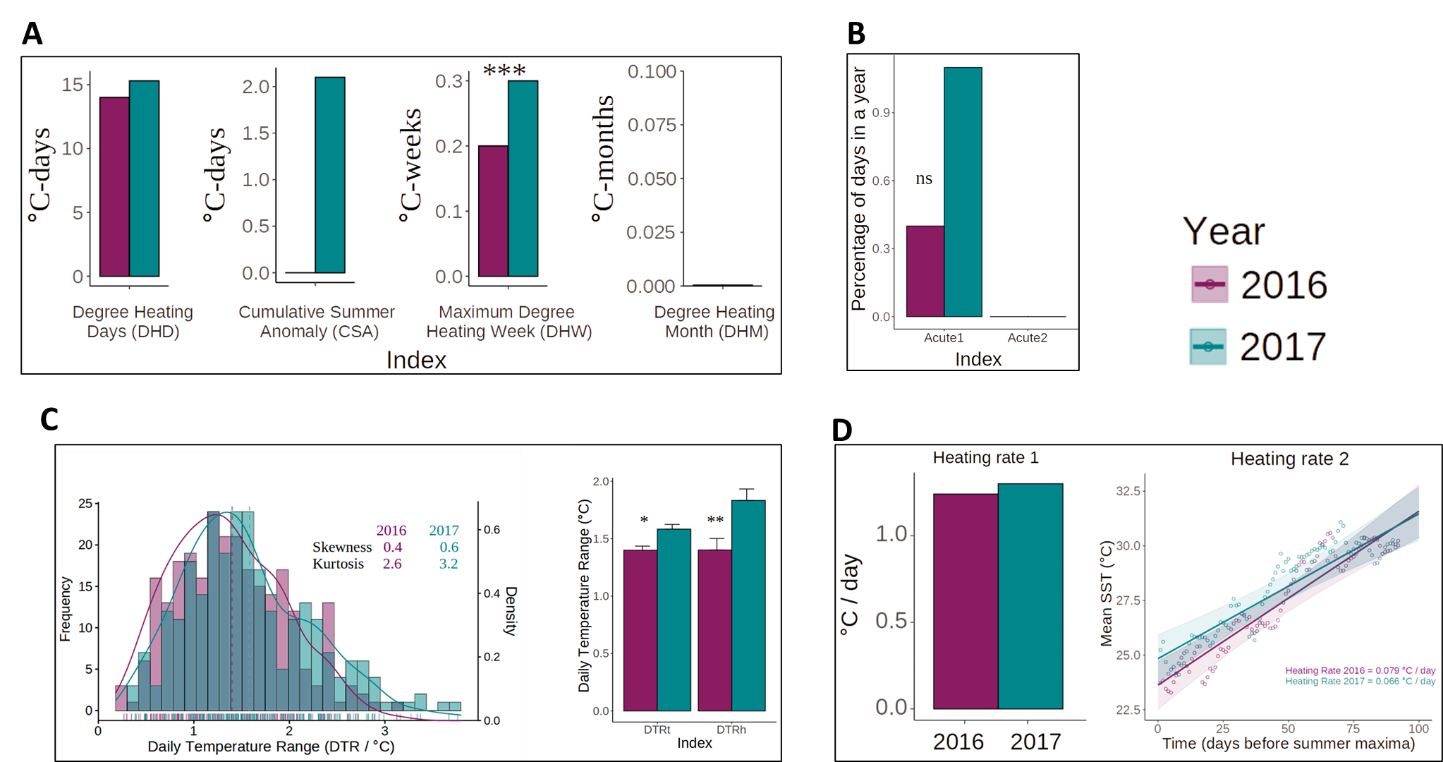
**

**Supplementary Figure S2.** (A) Temporal variations in cumulative thermal stress indices, (B) acute stress indices, (C) frequency distribution of daily DTR (daily temperature range) and Mean ± SE of DTR over the entire year and the warmest two months, and (D) heating rate. Asterisks represent significant differences between years or time periods (‘***’ P < 0.001, ‘**’ P < 0.01 ‘*’ P < 0.05 ‘·’ ‘ns’ P > 0.05).


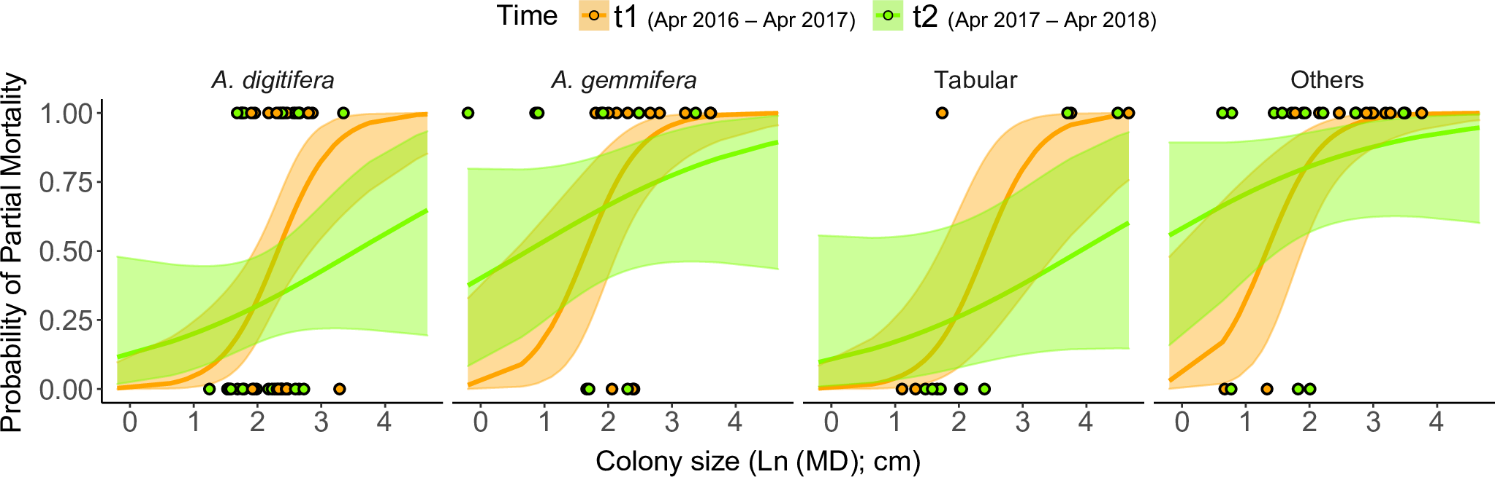


**Supplementary Figure S3.** Relationship between colony size and probability of partial mortality in different *Acropora* groups at time t1 and t2. Lines in the plot represent binomial regression fit with 95% confidence intervals. Dots in the plot represent the observed data.

**References**

Berkelmans R, De’ath G, Kininmonth S, Skirving WJ. 2004. A comparison of the 1998 and 2002 coral bleaching events on the Great Barrier Reef: Spatial correlation, patterns, and predictions. Coral Reefs. 23, 74-83. DOI:10.1007/s00338-003-0353-y

Chollett I, Enríquez S, and Mumby PJ. 2014. Redefining thermal regimes to design reserves for coral reefs in the face of climate change. PLoS ONE. 9, 1-8. DOI:10.1371/journal.pone.0110634.

Donner SD, Skirving WJ, Little, CM, Oppenheimer M, Hoegh-Guldberg O. 2005. Global assessment of coral bleaching and required rates of adaptation under climate change. Glob. Chang. Biol. 11, 2251-2265. DOI:10.1111/j.1365-2486.2005.01073.x

Maynard JA, Turner PJ, Anthony KRN, Baird AH, Berkelmans R, Eakin CM, et al. 2008. ReefTemp : An interactive monitoring system for coral bleaching using high-resolution SST and improved stress predictors. Geophys. Res. Lett. 35, L05603. DOI:10.1029/2007GL032175

Liu, G, Strong AE, Skirving W. 2003. Remote sensing of sea surface temperatures during 2002 Barrier Reef coral bleaching. *Eos, Trans. Am. Geophys. Union.* 84, 137-144. DOI:10.1029/2003EO150001.

Safaie A, Silbiger NJ, McClanahan TR, Pawlak G, Barshis DJ, Hench JL, et al. 2018. High frequency temperature variability reduces the risk of coral bleaching. Nat. Commun. 9, 1-12. DOI :10.1038/s41467-018-04074-2
